# Supplementary material for: Genome-wide haplotype-based association analysis of major depressive disorder in Generation Scotland and UK Biobank
Source: Transl Psychiatry. 2017 Nov 30;7:1263. doi: 10.1038/s41398-017-0010-9 (PMC5802488; doi:10.1038/s41398-017-0010-9)
Supplement: Supplementary file 1 — Supplementary Figure S1 [file 41398_2017_10_MOESM1_ESM.docx]

**
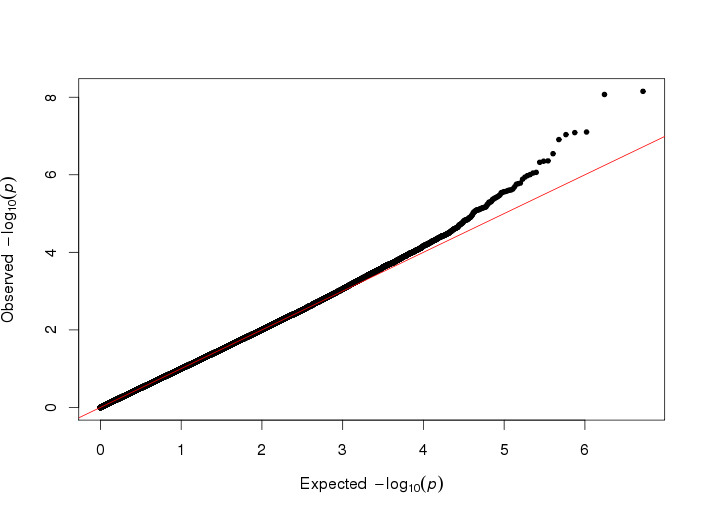
**

**Supplementary Figure S1.** Q-Q plot representing the –log_10_ *P*-values for an association between each assessed haplotype in the Generation Scotland: Scottish Family Health Study cohort and Major Depressive Disorder
