# Supplementary material for: Genome-wide haplotype-based association analysis of major depressive disorder in Generation Scotland and UK Biobank
Source: Transl Psychiatry. 2017 Nov 30;7:1263. doi: 10.1038/s41398-017-0010-9 (PMC5802488; doi:10.1038/s41398-017-0010-9)
Supplement: Supplementary file 2 — Supplementary Table 1 [file 41398_2017_10_MOESM2_ESM.doc]

**Supplementary Table 1.** The alleles that constitute the 12 haplotypes in the Generation Scotland: Scottish Family Health Study (GS:SFHS) cohort, where *P* < 10^-6^. Base pair (bp) positions are based on build GRCh37. Shaded areas represent haplotypes that were positioned within other haplotypes and comprised of the same alleles.

| Chr. 6 | | Chr. 7 | | Chr. 8 | | Chr. 10 | | Chr. 11 | | Chr. 12 | | Chr. 12 | | Chr. 15 | | Chr. 15 | |
| --- | --- | --- | --- | --- | --- | --- | --- | --- | --- | --- | --- | --- | --- | --- | --- | --- | --- |
| 108338267 – 108454437 bp | | 139682412 – 139708901 bp | | 79700362 – 80387861 bp | | 4588261 – 4822210 bp | | 2260854 – 2437425 bp | | 48159721 – 48263828 bp | | 116904503 – 117062860 bp | | 49206902 – 49260601 bp | | 93806477 – 93851224 bp | |
| rs17069173 | A | rs8192846 | A | rs11990466 | G | rs11814411 | A | rs11021859 | A | rs7135791 | A | rs16946695 | G | rs934741 | G | rs13313429 | A |
| rs4332013 | A | rs193946 | A | rs4739139 | G | rs387673 | G | rs11021861 | G | rs4760653 | A | rs1241224 | A | rs12593599 | G | rs11857333 | A |
| rs9386694 | A | rs10487666 | C | rs16906115 | G | rs416990 | A | rs7941585 | A | rs13632 | G | rs12830663 | A | rs12915772 | A | rs8033975 | A |
| rs9374002 | G | rs10487667 | C | rs6997891 | A | rs372744 | A | rs11021984 | G | rs7972177 | A | rs11068043 | C | rs7164451 | A | rs10520721 | A |
| rs9372177 | A | rs8192849 | C | rs3885022 | A | rs715987 | A | rs11821527 | A | rs11168236 | A | rs1241239 | G | rs1007662 | G | rs4777639 | G |
| rs9400180 | G | rs10251092 | G | rs17506083 | G | rs374802 | G | rs17737713 | A | rs2240108 | A | rs12372279 | G | rs4608275 | G | rs1542609 | G |
| rs2097525 | G | rs3801150 | A | rs2581738 | A | rs745782 | A | rs10831668 | G | rs6580637 | A | rs2707202 | A | rs4774529 | G | rs12148243 | A |
| rs9374007 | A | rs2267703 | G | rs12677936 | A | rs7906409 | A | rs4929971 | G | rs2301783 | A | rs4767413 | C | rs17384124 | A | rs17612678 | A |
| rs17069204 | A | rs4726499 | A | rs2587155 | G | rs10795187 | A | rs1011476 | C | rs12820931 | G | rs7316498 | G | rs7178573 | G | rs7170984 | A |
| rs12567 | A | rs7785584 | A | rs17506603 | A | rs12413638 | G | rs10765903 | A | rs17122326 | A | rs7306112 | A | rs10519199 | A | rs4777809 | A |
| rs1064346 | A | rs8192858 | C | rs4739140 | A | rs7089195 | A | rs4930074 | G | rs757344 | A | rs11068058 | G | rs11070664 | A | rs12438547 | A |
| rs9320250 | A | rs2267704 | G | rs2369537 | G | rs313500 | G | rs10831706 | A | rs1859281 | G | rs10774867 | G | rs11070665 | G | rs4777811 | A |
| rs9374013 | G | rs3801148 | A | rs4739111 | A | rs10904290 | A | rs7104786 | A | rs10747524 | G | rs4767418 | G | rs10519201 | C | rs11074155 | A |
| rs2027876 | G | rs8192859 | G | rs2010128 | G | rs2028690 | A | rs2521259 | A | rs12721364 | G | rs4767420 | G | rs12440434 | A | rs11074159 | A |
| rs6921166 | G | rs7795996 | A | rs11992182 | C | rs381443 | A | rs2521263 | G | rs2525046 | A | rs11068063 | G | rs2413923 | A | rs4777642 | A |
| rs2001144 | A | rs2284212 | G | rs1596434 | G | rs1008042 | A | rs1029409 | A | rs11574143 | G | rs7958181 | A | rs16952958 | A | rs4777812 | G |
| rs7769514 | A | rs2284213 | A | rs1858120 | G | rs1498271 | A | rs756919 | A | rs9729 | C | rs11610226 | C | rs7178085 | C | rs7167984 | A |
| rs7769535 | A | rs13221138 | G | rs10808826 | G | rs1498274 | A | rs7944004 | C | rs3847987 | C | rs11068069 | G | rs11638748 | G | rs16947747 | G |
| rs4946900 | G | rs2267705 | A | rs6987092 | A | rs569744 | G | rs7941928 | A | rs1544410 | G | rs10161436 | G | rs4491452 | A | rs16947760 | A |
| rs9374021 | G | rs2267706 | G | rs1443898 | A | rs11252607 | G | rs2651836 | A | rs7967152 | A | rs7133288 | A | rs10851473 | G | rs6497060 | A |
| rs12180499 | A | rs16882280 | A | rs12548956 | C | rs1443852 | A | rs2074019 | G | rs2239182 | A | rs4766793 | G | rs16961929 | G | rs4777828 | A |
| rs6919324 | G | rs17161334 | G | rs6993385 | G | rs11252615 | A | rs1501466 | A | rs2107301 | G | rs17581750 | G | rs11070668 | A | rs4777647 | C |
| rs17069274 | A | rs11761051 | A | rs1015028 | A | rs10904299 | A | rs2077078 | G | rs1540339 | G | rs4766796 | A | rs12915940 | G | rs10083565 | A |
| rs9486815 | G | rs17161335 | T | rs11995697 | G | rs12775823 | A | rs756923 | G | rs12721370 | C | rs11615308 | G | rs4775789 | A | rs13329166 | G |
| rs4245535 | G | rs17161337 | G | rs11996659 | G | rs1838238 | A | rs11022157 | C | rs886441 | G | rs7972894 | G | rs11070670 | A |  |  |
| rs218294 | A | rs740204 | G | rs10106730 | G | rs7096965 | G | rs2074022 | G | rs2189480 | C | rs12227287 | A | rs11636875 | A |  |  |
| rs218289 | A | rs12703488 | G | rs9283993 | A | rs10904300 | A | rs756926 | C |  |  | rs2393073 | A | rs8031813 | A |  |  |
|  |  |  |  | rs11782492 | A | rs11252622 | G | rs808963 | A |  |  | rs12813037 | A | rs4474633 | G |  |  |
|  |  |  |  | rs10448016 | A | rs17297024 | G | rs2521282 | G |  |  | rs11834175 | A |  |  |  |  |
|  |  |  |  | rs7000123 | C | rs10458787 | G | rs4930081 | G |  |  | rs11068094 | A |  |  |  |  |
|  |  |  |  | rs6999801 | G | rs1039197 | C | rs2651843 | G |  |  | rs10850677 | A |  |  |  |  |
|  |  |  |  | rs2120410 | A | rs11252631 | G | rs2521294 | A |  |  | rs4767422 | G |  |  |  |  |
|  |  |  |  | rs4739119 | C | rs6601853 | G | rs2651803 | A |  |  | rs10850679 | G |  |  |  |  |
|  |  |  |  | rs2067696 | A | rs313462 | G | rs800123 | A |  |  | rs2393074 | C |  |  |  |  |
|  |  |  |  | rs6990279 | A | rs313445 | A | rs10831846 | G |  |  | rs7314617 | A |  |  |  |  |
|  |  |  |  | rs4739148 | C | rs313438 | A | rs800153 | G |  |  | rs10850681 | A |  |  |  |  |
|  |  |  |  | rs11775912 | G | rs17298157 | G | rs808964 | A |  |  | rs11611974 | C |  |  |  |  |
|  |  |  |  | rs10098207 | C | rs313435 | G | rs708154 | G |  |  | rs17615703 | C |  |  |  |  |
|  |  |  |  | rs925246 | G | rs1353703 | A | rs708564 | A |  |  | rs11068102 | A |  |  |  |  |
|  |  |  |  | rs6983483 | A | rs2088500 | G | rs739673 | A |  |  | rs7958610 | A |  |  |  |  |
|  |  |  |  | rs11781378 | G | rs12255709 | A | rs2074234 | A |  |  | rs4766797 | A |  |  |  |  |
|  |  |  |  | rs17418058 | A | rs313451 | A | rs2301698 | C |  |  | rs7304254 | C |  |  |  |  |
|  |  |  |  | rs7003181 | C | rs313452 | C |  |  |  |  | rs2077158 | A |  |  |  |  |
|  |  |  |  | rs7842502 | G | rs313455 | G |  |  |  |  | rs11837475 | G |  |  |  |  |
|  |  |  |  | rs12680350 | A | rs2647337 | A |  |  |  |  | rs10850685 | G |  |  |  |  |
|  |  |  |  | rs12682560 | G | rs11598678 | C |  |  |  |  |  |  |  |  |  |  |
|  |  |  |  | rs6988545 | A | rs10508287 | A |  |  |  |  |  |  |  |  |  |  |
|  |  |  |  | rs17515867 | G | rs313430 | A |  |  |  |  |  |  |  |  |  |  |
|  |  |  |  | rs10464926 | A | rs167511 | A |  |  |  |  |  |  |  |  |  |  |
|  |  |  |  | rs10464927 | G | rs1838239 | G |  |  |  |  |  |  |  |  |  |  |
|  |  |  |  | rs12056707 | G | rs2165953 | G |  |  |  |  |  |  |  |  |  |  |
|  |  |  |  | rs7812820 | G | rs1391511 | G |  |  |  |  |  |  |  |  |  |  |
|  |  |  |  | rs1834743 | G | rs7916170 | G |  |  |  |  |  |  |  |  |  |  |
|  |  |  |  | rs10808828 | G | rs6601855 | G |  |  |  |  |  |  |  |  |  |  |
|  |  |  |  | rs7845577 | A | rs1498273 | A |  |  |  |  |  |  |  |  |  |  |
|  |  |  |  | rs539254 | A | rs7080649 | A |  |  |  |  |  |  |  |  |  |  |
|  |  |  |  | rs683929 | A | rs433430 | G |  |  |  |  |  |  |  |  |  |  |
|  |  |  |  | rs503431 | A | rs10904319 | A |  |  |  |  |  |  |  |  |  |  |
|  |  |  |  | rs17517571 | G | rs10904321 | G |  |  |  |  |  |  |  |  |  |  |
|  |  |  |  | rs558228 | A | rs7068675 | C |  |  |  |  |  |  |  |  |  |  |
|  |  |  |  | rs519121 | G | rs10904323 | G |  |  |  |  |  |  |  |  |  |  |
|  |  |  |  | rs10504690 | G | rs11252672 | G |  |  |  |  |  |  |  |  |  |  |
|  |  |  |  | rs16906486 | A | rs11252674 | G |  |  |  |  |  |  |  |  |  |  |
|  |  |  |  | rs16906512 | G | rs11252679 | A |  |  |  |  |  |  |  |  |  |  |
|  |  |  |  | rs5003440 | A | rs17133449 | G |  |  |  |  |  |  |  |  |  |  |
|  |  |  |  | rs2222361 | G | rs2397839 | A |  |  |  |  |  |  |  |  |  |  |
|  |  |  |  | rs16906530 | A | rs10904328 | G |  |  |  |  |  |  |  |  |  |  |
|  |  |  |  | rs12682020 | G | rs11252688 | A |  |  |  |  |  |  |  |  |  |  |
|  |  |  |  | rs10504696 | G | rs2123348 | G |  |  |  |  |  |  |  |  |  |  |
|  |  |  |  | rs7000109 | A | rs17372054 | G |  |  |  |  |  |  |  |  |  |  |
|  |  |  |  | rs16906568 | A | rs12251644 | C |  |  |  |  |  |  |  |  |  |  |
|  |  |  |  | rs16906578 | A | rs11596789 | A |  |  |  |  |  |  |  |  |  |  |
|  |  |  |  | rs10108874 | G | rs10508288 | A |  |  |  |  |  |  |  |  |  |  |
|  |  |  |  | rs1551712 | A | rs4146653 | A |  |  |  |  |  |  |  |  |  |  |
|  |  |  |  | rs16906594 | G | rs7899916 | G |  |  |  |  |  |  |  |  |  |  |
|  |  |  |  | rs10504699 | A | rs10904332 | C |  |  |  |  |  |  |  |  |  |  |
|  |  |  |  | rs6473142 | G | rs4881340 | A |  |  |  |  |  |  |  |  |  |  |
|  |  |  |  | rs1227646 | G | rs10904333 | G |  |  |  |  |  |  |  |  |  |  |
|  |  |  |  | rs1227630 | A | rs7896112 | G |  |  |  |  |  |  |  |  |  |  |
|  |  |  |  | rs1227632 | A | rs12357812 | A |  |  |  |  |  |  |  |  |  |  |
|  |  |  |  | rs1227634 | A | rs2894969 | G |  |  |  |  |  |  |  |  |  |  |
|  |  |  |  | rs1915554 | A | rs11252715 | G |  |  |  |  |  |  |  |  |  |  |
|  |  |  |  | rs1234900 | G | rs11252717 | G |  |  |  |  |  |  |  |  |  |  |
|  |  |  |  | rs1227655 | G | rs10508290 | G |  |  |  |  |  |  |  |  |  |  |
|  |  |  |  | rs16906665 | A | rs1869210 | A |  |  |  |  |  |  |  |  |  |  |
|  |  |  |  | rs1227649 | G | rs17133585 | A |  |  |  |  |  |  |  |  |  |  |
|  |  |  |  | rs1870575 | A | rs7893291 | C |  |  |  |  |  |  |  |  |  |  |
|  |  |  |  | rs7817332 | G | rs11818567 | A |  |  |  |  |  |  |  |  |  |  |
|  |  |  |  | rs10504700 | A | rs1901632 | A |  |  |  |  |  |  |  |  |  |  |
|  |  |  |  | rs10504702 | G | rs7900146 | A |  |  |  |  |  |  |  |  |  |  |
|  |  |  |  | rs7829769 | A | rs9423544 | A |  |  |  |  |  |  |  |  |  |  |
|  |  |  |  | rs1531346 | A | rs7082636 | A |  |  |  |  |  |  |  |  |  |  |
|  |  |  |  | rs1460162 | A |  |  |  |  |  |  |  |  |  |  |  |  |
|  |  |  |  | rs10504705 | G |  |  |  |  |  |  |  |  |  |  |  |  |
|  |  |  |  | rs1380634 | A |  |  |  |  |  |  |  |  |  |  |  |  |
|  |  |  |  | rs1350681 | A |  |  |  |  |  |  |  |  |  |  |  |  |
|  |  |  |  | rs1460168 | G |  |  |  |  |  |  |  |  |  |  |  |  |
|  |  |  |  | rs16906775 | G |  |  |  |  |  |  |  |  |  |  |  |  |
|  |  |  |  | rs4129499 | C |  |  |  |  |  |  |  |  |  |  |  |  |
|  |  |  |  | rs4074500 | G |  |  |  |  |  |  |  |  |  |  |  |  |
|  |  |  |  | rs11778785 | C |  |  |  |  |  |  |  |  |  |  |  |  |
|  |  |  |  | rs4739607 | G |  |  |  |  |  |  |  |  |  |  |  |  |
|  |  |  |  | rs4074079 | C |  |  |  |  |  |  |  |  |  |  |  |  |
|  |  |  |  | rs7462026 | G |  |  |  |  |  |  |  |  |  |  |  |  |
|  |  |  |  | rs7462051 | G |  |  |  |  |  |  |  |  |  |  |  |  |
|  |  |  |  | rs6999948 | G |  |  |  |  |  |  |  |  |  |  |  |  |
|  |  |  |  | rs9643438 | G |  |  |  |  |  |  |  |  |  |  |  |  |
|  |  |  |  | rs10957934 | A |  |  |  |  |  |  |  |  |  |  |  |  |
|  |  |  |  | rs11985579 | C |  |  |  |  |  |  |  |  |  |  |  |  |
|  |  |  |  | rs12678425 | G |  |  |  |  |  |  |  |  |  |  |  |  |
|  |  |  |  | rs7462145 | G |  |  |  |  |  |  |  |  |  |  |  |  |
|  |  |  |  | rs12545192 | A |  |  |  |  |  |  |  |  |  |  |  |  |
|  |  |  |  | rs6998419 | A |  |  |  |  |  |  |  |  |  |  |  |  |
|  |  |  |  | rs10086740 | G |  |  |  |  |  |  |  |  |  |  |  |  |
|  |  |  |  | rs11991397 | C |  |  |  |  |  |  |  |  |  |  |  |  |
|  |  |  |  | rs7465051 | A |  |  |  |  |  |  |  |  |  |  |  |  |
|  |  |  |  | rs7461233 | A |  |  |  |  |  |  |  |  |  |  |  |  |
|  |  |  |  | rs7836491 | C |  |  |  |  |  |  |  |  |  |  |  |  |
|  |  |  |  | rs7012426 | G |  |  |  |  |  |  |  |  |  |  |  |  |
|  |  |  |  | rs7460206 | G |  |  |  |  |  |  |  |  |  |  |  |  |
|  |  |  |  | rs9650266 | A |  |  |  |  |  |  |  |  |  |  |  |  |
|  |  |  |  | rs6473158 | A |  |  |  |  |  |  |  |  |  |  |  |  |
|  |  |  |  | rs10089772 | G |  |  |  |  |  |  |  |  |  |  |  |  |
|  |  |  |  | rs7836445 | G |  |  |  |  |  |  |  |  |  |  |  |  |
|  |  |  |  | rs11777412 | G |  |  |  |  |  |  |  |  |  |  |  |  |
